# Long term analysis of microbiological isolates and antibiotic susceptibilities in acute-onset postoperative endophthalmitis: a UK multicentre study

**DOI:** 10.1038/s41433-025-03673-w

**Published:** 2025-02-12

**Authors:** Boon Lin Teh, Ariel Yuhan Ong, Ankur Mehta, Vy Hoang, Chris Settle, Andrew J. Lotery, Peter Charbel Issa, Jonathan Smith, David H. Steel

**Affiliations:** 1 Sunderland Eye Infirmary, Sunderland, United Kingdom; 2 Oxford Eye Hospital, Oxford, United Kingdom; 3 Moorfields Eye Hospital, London, United Kingdom; 4 Institute of Ophthalmology, University College London, London, United Kingdom; 5 Southampton Eye Unit, University Hospital Southampton, Southampton, United Kingdom; 6 Department of Microbiology, South Tyneside and Sunderland NHS Foundation Trust, South Tyneside, United Kingdom; 7 Faculty of Medicine, University of Southampton, Southampton, United Kingdom; 8 Nuffield Laboratory of Ophthalmology, Nuffield Department of Clinical Neurosciences, University of Oxford, Oxford, United Kingdom; 9 Department of Ophthalmology, TUM University Hospital, School of Medicine and Health, Technical University of Munich (TUM), Munich, Germany; 10 Biosciences Institute, Newcastle University, Newcastle upon Tyne, United Kingdom

**Keywords:** Microbiology, Eye diseases

## Abstract

**Objectives:**

To review the trend of microbial isolates for postoperative endophthalmitis (POE) in the United Kingdom (UK) and determine the sensitivity to current empirical intravitreal antibiotic treatment.

**Methods:**

We conducted a long term multicentre consecutive case review of POE across 3 geographically distant tertiary eye centres in the UK: Sunderland Eye Infirmary (2000–2022), Oxford Eye Hospital (2016–2022), and Southampton General Hospital (2016–2022). Data on the microbial samples taken and results including sensitivities to antibiotics agents given were collected. Poisson regression was used to analyse microbial trends and outcomes were considered statistically significant at a level of *p* < 0.05.

**Results:**

179 consecutive eyes of 177 patients with POE met our inclusion criteria. The most common primary procedure was phacoemulsification and IOL insertion followed by intravitreal injections. 104 (58.1%) were culture positive and most were Gram-positive bacteria (85, 81.7%). The microbial trend consistently showed *Staphylococcus epidermidis* and unspecified coagulase-negative *Staphylococci* to be the most prevalent pathogens. Poisson regression showed no statistically significant change in any of the bacterial isolates over our study period. Antibiotic sensitivity data was available for 74% of the culture positive samples (77/104). All Gram-positive bacteria (68/68, 100%) and most (8/9, 88.9%) Gram-negative bacteria were sensitive to the empirical antibiotics (Vancomycin and Ceftazidime/Amikacin) given at presentation.

**Conclusions:**

Most of the bacterial isolates causing POE in the UK are Gram-positive bacteria, and the trend has remained stable over more than two decades. Current empirical treatment with intravitreal Vancomycin and Ceftazidime/Amikacin provides effective broad coverage for the vast majority of cases.

## Introduction

Postoperative endophthalmitis (POE) is a rare sight-threatening complication that may occur following any intraocular procedure. It is characterised by severe inflammation of the inner layers of the eye secondary to infection. The severity and clinical course of POE depend on the type and virulence of inoculated pathogens, as well as the time to diagnosis and treatment [1, 2].

Over the last 20 years, there has been a reduction in the incidence of POE following cataract surgery, thought to be associated with the consistent use of antiseptics and the introduction of intracameral antibiotics [3–6]. However, at the same time, with the success of anti-vascular endothelial growth factor (VEGF) treatments for a range of common retinal diseases, there has been a rapid increase in the number of intravitreal injections delivered over the past two decades [7, 8]. Whilst the overall incidence of POE has remained low, the sheer increase in number of injections performed has made it one of the major causes of POE [9, 10]. Reassuringly, a recent study reported a substantial reduction in the incidence of POE following any intraocular procedure by nearly 75% over the past two decades [11].

The current management of POE is mainly informed by the landmark Endophthalmitis Vitrectomy Study (EVS) [12], reinforced by guidelines from learned societies [13]. Vitreous and/or aqueous samples were first obtained for microbiologic testing, followed by empirical intravitreal injection of antibiotics. At present, the most commonly used antibiotic combination is Vancomycin and Ceftazidime or Amikacin [14]. However, there is growing concern regarding increasing bacterial resistance to antibiotics globally [15, 16] and this might also impact on the optimal management of POE.

In this study, we aimed to review the trend of microbial isolates for POE and determine the sensitivity to current empirical intravitreal antibiotic treatment using data from 3 large tertiary ophthalmic units in the United Kingdom (UK).

## Methods

This was a multicentre study involving 3 geographically distant tertiary eye centres in the UK: Sunderland Eye Infirmary, Oxford Eye Hospital and Southampton General Hospital. The study was classed as a continuous departmental audit and surveillance as part of clinical governance requirements, and was therefore exempt from requiring formal ethics approval.

A database was established in January 2000 in Sunderland Eye Infirmary to prospectively record all patients with endophthalmitis presenting to the unit. Data was collected from this database up to 31^st^ July 2022. In the other two units (Oxford Eye Hospital and Southampton General Hospital), retrospective review of all patients presenting with endophthalmitis were conducted from 1^st^ January 2016 to 31^st^ July 2022 using departmental electronic medical record systems or paper records.

We included all consecutive patients presenting with acute-onset POE presenting within 6 weeks of any primary intraocular procedure. The initial diagnosis was made clinically, based on signs and symptoms associated with endophthalmitis such as pain, redness, loss of vision, hypopyon, fibrin and vitritis, supported by adjunctive B-scan ultrasonography. Patients with delayed-onset endophthalmitis (more than 6 weeks after primary intraocular procedure), endogenous endophthalmitis and those associated with trauma were excluded [10].

Data collected included patient demographics, details of the primary ophthalmic procedure, timing of presentation, microbiological samples taken and results including sensitivities to antimicrobial agents given, initial management, and visual acuity (VA) at presentation and final follow-up (at least 3 months following acute-onset POE).

Across all 3 units, initial management of POE was either a ‘tap and inject’ procedure (obtaining samples of intraocular fluids followed by intravitreal antibiotic injection) or patients proceeding directly to pars plana vitrectomy with intravitreal antibiotic cover. For tap and inject, aqueous samples were obtained through peripheral clear cornea paracentesis using 30-gauge needle attached to a 1 ml plastic sterile disposable tuberculin syringe inserted parallel to the iris plane. Vitreous samples were taken via vitreous taps with needle aspiration using a 23- or 25-gauge needle, or mechanically via vitreous cutter, depending on local protocols. The intravitreal antibiotics of choice were Vancomycin (1–2 mg/0.1 mL) and Ceftazidime (2 mg/0.1 mL). Intravitreal Amikacin (0.4 mg/0.1 mL) was used in place of Ceftazidime in those allergic to penicillin or cephalosporins, depending on local treatment protocols. Choice of topical antibiotics, steroids, cycloplegics, and oral antibiotics and steroids were prescribed at the discretion of the treating clinicians.

All data collected were anonymised and recorded in a standardised Microsoft Excel spreadsheet (Microsoft Corporation, Redmond, WA). VA values were converted to the appropriate logarithm of the minimum angle of resolution (logMAR) equivalent for analyses. VA of counting fingers (CF), hand movements (HM), perception of light (PL), and no perception of light (NPL) were assigned logMAR values of 2.1, 2.4, 2.7, and 3.0 respectively, as per the National Ophthalmology Database audit [17].

Descriptive data were presented using tabular and graphical summaries. Statistical analyses were performed using IBM SPSS Statistics, version 29 (IBM Corporation, Armonk, NY, USA). Outcomes were considered statistically significant at a level of *p* < 0.05. Quantitative variables were presented as frequencies with percentages. Non-parametric continuous variables were reported as median, interquartile range (IQR) and range. The Chi-squared test was used to evaluate associations between categorical variables. We expressed results using odds ratios (OR) and their 95% confidence intervals (CI). Multivariate analysis by stepwise logistic regression was used to examine variables associated with a poor visual outcome (defined as logMAR 1.0 or worse), and Poisson regression was used to analyse microbial trends.

## Results

We identified a total of 179 eyes of 177 patients with POE that met our inclusion criteria (106 eyes of 104 patients from Sunderland Eye Infirmary, 35 from Oxford Eye Hospital and 38 from Southampton General Hospital), following exclusion of 6 cases of delayed-onset endophthalmitis, 5 trauma-related endophthalmitis and 15 endogenous endophthalmitis.

Demographic details of patients in our cohort are summarised in Table 1. The median age was 76.8 years (range 27–94), with slight female preponderance (53.7%). The median presenting VA was 2.40 logMAR (or HM).

**Table 1:** Patient demographics.

Patient demographics	*N* = 179
Female. *N* (%)	96 (53.7%)
Age, years. Median (IQR)	76.8 (69–82)
Days since primary procedure. Median (IQR)	6 (3–14)
Presenting logMAR visual acuity. Median (IQR)	2.4 (1.0–2.4)
Positive culture. *N* (%)	104 (58.1%)
Final logMAR visual acuity. Median (IQR)	0.8 (0.3–2.1)

*IQR* interquartile range, *logMAR* logarithm of the minimum angle of resolution.

The primary ophthalmic intervention is shown in Table 2. The most common primary procedure was phacoemulsification and IOL insertion comprising 89 (49.7%) of cases followed by intravitreal injections (67, 37.4%). The aetiology of POE over the course of the study is illustrated in Supplementary Fig. 1.

**Table 2:** Primary ophthalmic procedure.

Primary ophthalmic procedure	Number (%)
Phacoemulsification with IOL implant	89 (49.7)
Intravitreal injection	67 (37.4)
Vitrectomy	14 (7.8)
Trabeculectomy	5 (2.8)
Glaucoma drainage implant	1 (0.6)
Corneal graft	1 (0.6)
Secondary IOL	1 (0.6)
Squint surgery	1 (0.6)
Total	179 (100)

*IOL* intraocular lens.

All patients underwent aqueous and/or vitreous sampling as part of their initial management before intravitreal injection of antibiotics. The majority (161/179, 89.9%) underwent a tap and inject procedure at presentation, while the remainder (18/179, 10.1%) underwent initial pars plana vitrectomy. The microbiological sampling technique used is shown in Supplementary Table 1. Treating clinicians have aimed to obtain vitreous samples from all eyes. However, this was not possible in 10 eyes due to reasons such as dry vitreous tap or silicone oil-filled vitreous cavity; consequently, only anterior chamber (AC) taps were successfully performed in these cases. All samples were sent for microbiological testing which included microscopy and gram staining as well as subsequent culture and sensitivities of identified microbes.

Of all the samples sent, 104 (58.1%) were culture positive. The majority of the cultures were of a single type of bacterium (94/104; 90.4%) and the remaining 10 (9.6%) grew more than one type. Most of the culture positive cases were Gram-positive bacteria (85, 81.7%). Subgroup analysis showed no statistically significant difference across the three centres (*p* = 0.87; Supplementary Table 2). Table 3 outlines the microbiological profile of the culture specimens.

**Table 3:** Microbiological profile of culture positive cases.

Microbes	Number (%)
Monomicrobial (*n* = 94)	
*Staphylococcus epidermidis*	19 (18.3%)
Unspecified coagulase-negative *Staphylococci*	19 (18.3%)
*Enterococcus faecalis*	11 (10.6%)
*Pseudomonas aeruginosa*	9 (8.7%)
*Staphylococcus aureus*	7 (6.7%)
*Haemophilus influenzae*	4 (3.8%)
*Serratia marcescens*	3 (2.9%)
*Streptococcus oralis*	3 (2.9%)
*Streptococcus pneumoniae*	3 (2.9%)
*Streptococcus salivarius*	2 (1.9%)
*Moraxella* spp.	2 (1.9%)
*Gram positive cocci*	2 (1.9%)
*Streptococcus mitis*	1 (1.0%)
*Staphylococcus capitis*	1 (1.0%)
*Streptococcus parasanguinis*	1 (1.0%)
*Staphylococcus warneri*	1 (1.0%)
*Streptococcus anginosus*	1 (1.0%)
Alpha haemolytic *Streptococcus*	1 (1.0%)
*Gram positive bacilli=*	1 (1.0%)
*Granulicatella adiacens*	1 (1.0%)
*Micrococcus luteus*	1 (1.0%)
*Neisseria* spp.	1 (1.0%)
Polymicrobial (*n* = 10)	
*Staphylococcus epidermidis* and *Staphylococcus hominis* and *Streptococcus mitis*	2 (1.9%)
*Brevibacterium casei* and *Staphyloccocus aureus*	1 (1.0%)
Gram positive cocci and unspecified coagulase-negative *Staphylococci*	1 (1.0%)
*Micrococcus* and unspecified coagulase-negative *Staphylococci*	1 (1.0%)
*Streptococcus sanguis and Staphylococcus aureus*	1 (1.0%)
*Ralstonia picketti* and alpha haemolytic *Streptococcus*	1 (1.0%)
*Staphylococcus epidermidis* and unspecified coagulase-negative *Staphylococci*	1 (1.0%)
*Staphylococcus c*aprae and *Staphylococcus hominis* and *Staphylococcus epidermidis*	1 (1.0%)
*Staphylococcus warneri* and *Enterococcus faecalis*	1 (1.0%)

**Fig. 1:**
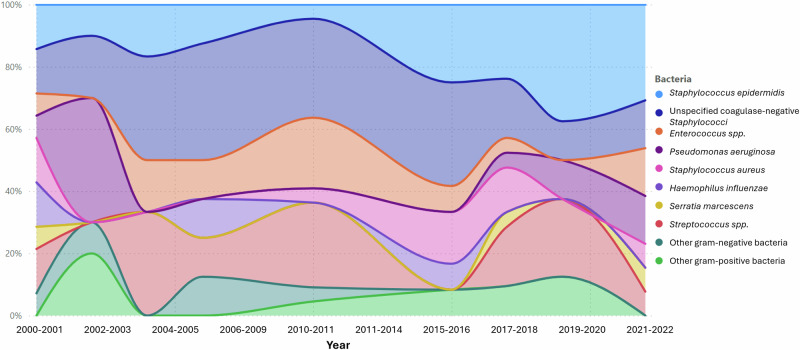
Area plot showing the trends of pathogens causing acute postoperative endophthalmitis (POE) in 104 culture positive cases.

The microbial trend over more than two decades consistently showed *Staphylococcus epidermidis* and unspecified coagulase-negative *Staphylococci* to be the most prevalent pathogens (Fig. 1). Poisson regression showed no statistically significant change in any of the bacterial isolates over our study period. (Supplementary Table 3).

The microbial yield of the aqueous samples was significantly lower at 32.4% compared to 55.6% in vitreous samples (*p* < 0.05). Adding an aqueous sample on top of the vitreous sample marginally improved the microbial yield to 58.1% (*p* = 0.64), (Supplementary Table 4). Antibiotic sensitivity data was not available for a quarter of the culture positive samples (27/104, 26.0%). For the remaining 77 samples, all Gram-positive bacteria (68/68, 100%) and most (8/9, 88.9%) Gram-negative bacteria were sensitive to the empirical antibiotics given at presentation. One eye grew Gram-negative *Serratia marcescens* which was resistant to these antibiotics and proceeded to vitrectomy. Nonetheless, the eye developed recurrent retinal detachments requiring multiple vitrectomies and subsequently became phthisical with NPL vision. Overall, 98.7% (76/77) of our bacterial isolates were susceptible to Vancomycin and Ceftazidime/Amikacin.

Two patients in our series had bilateral endophthalmitis. One had same day bilateral intravitreal anti-VEGF injection for diabetic macular oedema. Samples from both eyes had polymicrobial growth of *Staphylococcus epidermidis*, *Staphylococcus hominis* and *Streptococcus mitis*. Another patient had sequential cataract surgery 5 months apart and unfortunately developed POE in both eyes. The first eye grew *Streptococcus pneumoniae* and the second eye was culture negative. Nasolacrimal duct obstruction was identified as a potential risk factor for the patient developing bilateral sequential POE.

Overall, median VA at final follow up was 0.8 logMAR (Snellen equivalent 6/38), with 40.1% eyes having 1.0 logMAR (Snellen equivalent 6/60) vision or worse. Univariable analysis demonstrated that poor presenting visual acuity (*p* < 0.001) and positive microbiological culture (*p* = 0.002) were significantly associated with a poor visual outcome (Supplementary Table 5). When we performed multivariable logistic regression, we found that presenting VA (OR 4.69, 95% CI 2.13–10.33) was the only statistically significant predictor for a poor visual outcome (*p* < 0.001), after adjusting for other variables including age, gender, primary ophthalmic procedure, days since primary procedure, microbiological yield (culture positive), Gram stain results, presence of multiple bacterial strains and antibiotics susceptibility (Supplementary Table 6).

## Discussion

Antimicrobial resistance (AMR) has been identified as one of the most pressing global challenges with the World Health Organisation (WHO) declaring AMR as one of the top 10 global public health threats [18]. This is also relevant in management of endophthalmitis as there are limited antibiotic alternatives to Vancomycin and Ceftazidime known to be safe for intraocular use [19]. In our study of 77 culture positive eyes with antibiotic sensitivity data, all Gram-positive bacteria (68/68, 100%) were sensitive to Vancomycin and 8 of the 9 (88.9%) Gram-negative bacteria were sensitive to Ceftazidime/Amikacin. Chen et al. [20] recently summarised the published literature of bacterial isolates and their in-vitro antibiotics susceptibility globally. While Vancomycin has excellent coverage for Gram-positive bacteria with around 96–100% susceptibility, Ceftazidime and Amikacin were reported to have much lower susceptibility, ranging from 38 to 100% and 64 to 100% respectively for Gram-negative bacteria. Though our study showed excellent antibiotic susceptibility for bacterial isolates in our cohort, there is a need for continuous monitoring of antibiotic resistance patterns and explore other potential antibiotic alternatives to treat POE.

The management of endophthalmitis relies on the initiation of appropriate empirical antibiotic therapy that targets the most common causative organisms. It is therefore important to understand the regional microbial spectrum as it has been recognised that there is geographical variation in the microbial profile of POE [21]. In our study, the spectrum of identified microbes showed that 81.7% of culture positive cases were due to Gram-positive bacteria, with coagulase-negative *Staphylococci* (mostly *Staphylococcus epidermidis*) being the most prevalent species causing POE. This is largely similar to other USA, European and Australian studies. The EVS study [12] conducted in the USA in 1995 showed that 94% of the bacterial isolates were Gram-positive with 70% of them being coagulase-negative *Staphylococci*. Similarly, in a multicentre European study conducted by the European Society of Cataract & Refractive Surgeons (ESCRS) in 2007, all culture-positive endophthalmitis cases were due to Gram-positive bacteria, with 40% being *Staphylococcus epidermidis* [22]. Moloney et al. in 2014 reported on their 15-year results of culture-proven endophthalmitis in Australia where around 85% were Gram-positive organisms [23].

In contrast, a review over two decades in India has shown that Gram-positive organisms accounted for a much lower proportion for POE, varying from 37.6% to 64.8% [24]. Another study in North-East India also reported that only around 46% of their exogenous endophthalmitis cases were caused by Gram-positive organisms, and the majority (27%) were in fact due to Gram-negative *Pseudomonas* species [25]. The POE isolates and their antibiotic sensitivity from different countries/regions is summarised in Table 4.

**Table 4:** Summary of postoperative bacterial endophthalmitis isolates from different countries/regions, along with their sensitivity to empirical intravitreal antibiotic treatment.

Study period	Group	Country/Region	Gram-positive bacteria	Gram-negative bacteria	Gram-positive bacteria sensitivity to Vancomycin	Gram-negative bacteria sensitivity to Ceftazidime/Amikacin
1990–1994	EVS [12]	USA	94.2%	5.8%	100%	89.5%
1991–2015	Joseph et al. [27]	India	67.7%	32.3%	96%	38–69%
1996–2019	Chen et al. [20]	Taiwan	49.9%	49.6%	99.8%	93.7–96.9%
1998–2013	Moloney et al. [23]	Australia	84.5%	15.5%	100%	100%
2000–2022	Our study	UK	81.7%	18.3%	100%	88.9%
2002–2011	Schimel et al. [26]	USA	87.2%	12.8%	100%	100%
2003–2006	ESCRS study [22]	Europe	100%	0%	100%	–

*EVS* Endophthalmitis Vitrectomy Study, *ESCRS* European Society of Cataract and Refractive Surgeons.

In terms of microbial trends, we found that the microbial profile of culture positive POE cases has remained largely stable within the three geographically distant UK centres, with no major shifts in bacterial isolates. Our findings align with similar studies conducted internationally. A 10-year review in the USA [26] and a 25-year study in India [27] both reported stable microbial profiles over their respective study periods. This suggests a potential consistency in the types of bacteria associated with POE within individual geographical regions.

Our study showed that phacoemulsification and IOL implant accounted for nearly half of the POE in our cohort (49.7%), followed by intravitreal injections (37.4%). Nevertheless, there has been a change in trend with intravitreal injections overtaking phacoemulsification as the leading cause of POE in recent years (Supplementary Fig. 1), agreeing with other published reports [28, 29].

Two patients in our series had bilateral endophthalmitis. One patient was known to be diabetic and another had nasolacrimal duct obstruction, both of which were recognised risk factors for POE [6]. Previous study also suggested a potential underlying genetic predisposition increasing individual patients’ risks of developing endophthalmitis [30].

We found that our microbial yield from vitreous samples was significantly higher than aqueous samples. This is consistent with multiple studies highlighting the superiority of vitreous over aqueous fluid in improving microbiological yield [31–33]. In our cohort, we managed to obtain vitreous samples from the majority of the cases (169/179, 94.4%). Overall, 58.1% of our POE cohort had positive microbial cultures, similar to previous published UK studies [31, 34].

The issue of a high culture-negative rate could potentially be addressed with the advancement in diagnostic technologies, such as polymerase chain reaction (PCR) testing, metagenomic and whole genome sequencing (WGS) [35, 36]. These methods allow for direct, large-scale parallel sequencing of single DNA molecules, providing detailed identification of pathogens from intraocular fluid samples. This enhanced capability allows for a timely and accurate diagnosis of endophthalmitis, facilitating more targeted treatment strategies. Lee et al. [36] in their prospective cohort study demonstrated that WGS has helped to identify potential pathogens in 33% of their culture-negative cases. It was also reported that higher bacterial DNA load other than *Staphylococcus epidermidis* is associated with worse outcomes, suggesting this could function as a prognostic marker. Despite the advantages, PCR assays can be limited by their inability to distinguish contaminants from true infections, leading to ambiguous results [37]. A recent review also found that these techniques are not always readily available, require advanced bioinformatic analysis and are expensive [38]. Further research is therefore needed to ascertain the optimal approach in incorporating these new technologies to complement the diagnostic process.

Although obtaining intraocular fluid samples for microbiologic testing has conventionally been a standard part of initial management of POE, the necessity for sampling has recently been contended with studies showing culture results having limited effect on subsequent clinical management [39, 40]. We intend to explore this interesting controversy in more detail in a separate study.

Our study has several limitations inherent to retrospective analyses that could affect the robustness of the outcomes and conclusions. The quality and completeness of the documented information were variable, particularly in the earlier chronological cases from the early 2000s. Some original records also no longer exist, making further analysis impossible in these instances. Additionally, we only included data from centres in the UK, which may limit the generalisability of our findings to other countries with potential differences in the spectrum of causative pathogens. Nevertheless, we believe that the large numbers of patients from 3 separate and geographically distant tertiary eye centres across the UK provides a robust overview of the state of play for POE in this region.

## Conclusions

In summary, we provide a comprehensive analysis of the microbial profile of acute-onset POE and the antibiotic sensitivity patterns across three geographically distant UK tertiary eye centres over an extended period. The stability in the trend of our POE pathogens, coupled with their excellent antibiotic susceptibility, suggests that current empirical treatment with intravitreal Vancomycin and Ceftazidime/Amikacin provides effective broad coverage for the vast majority of cases. It is likely that this antibiotic combination will remain relevant in the future, assuming no rapid development of antibiotic resistance. Continuous surveillance of POE trends would help to identify any shifts in microbial profiles or resistance patterns.

## Summary

### What was known before

- There is geographical variation in pathogens causing postoperative endophthalmitis with growing concerns of antimicrobial resistance especially in Gram-negative bacteria.

### What this study adds

- Majority of bacterial isolates causing postoperative endophthalmitis in the UK are Gram-positive bacteria with no major shifts in the spectrum of causative pathogens.
- Empirical treatment using intravitreal Vancomycin and Ceftazidime/Amikacin remained effective with low levels of resistance in our cohort.

## Supplementary information

Supplementary Figure 1

Supplementary Table 1

Supplementary Table 2

Supplementary Table 3

Supplementary Table 4

Supplementary Table 5

Supplementary Table 6

## Data Availability

The datasets generated during and/or analysed during the current study are available from the corresponding author on reasonable request.
