# Supplementary figures and images for: Long term analysis of microbiological isolates and antibiotic susceptibilities in acute-onset postoperative endophthalmitis: a UK multicentre study
Source: Eye (Lond). 2025 Feb 12;39(8):1470–5. doi: 10.1038/s41433-025-03673-w (PMC12089534; doi:10.1038/s41433-025-03673-w)

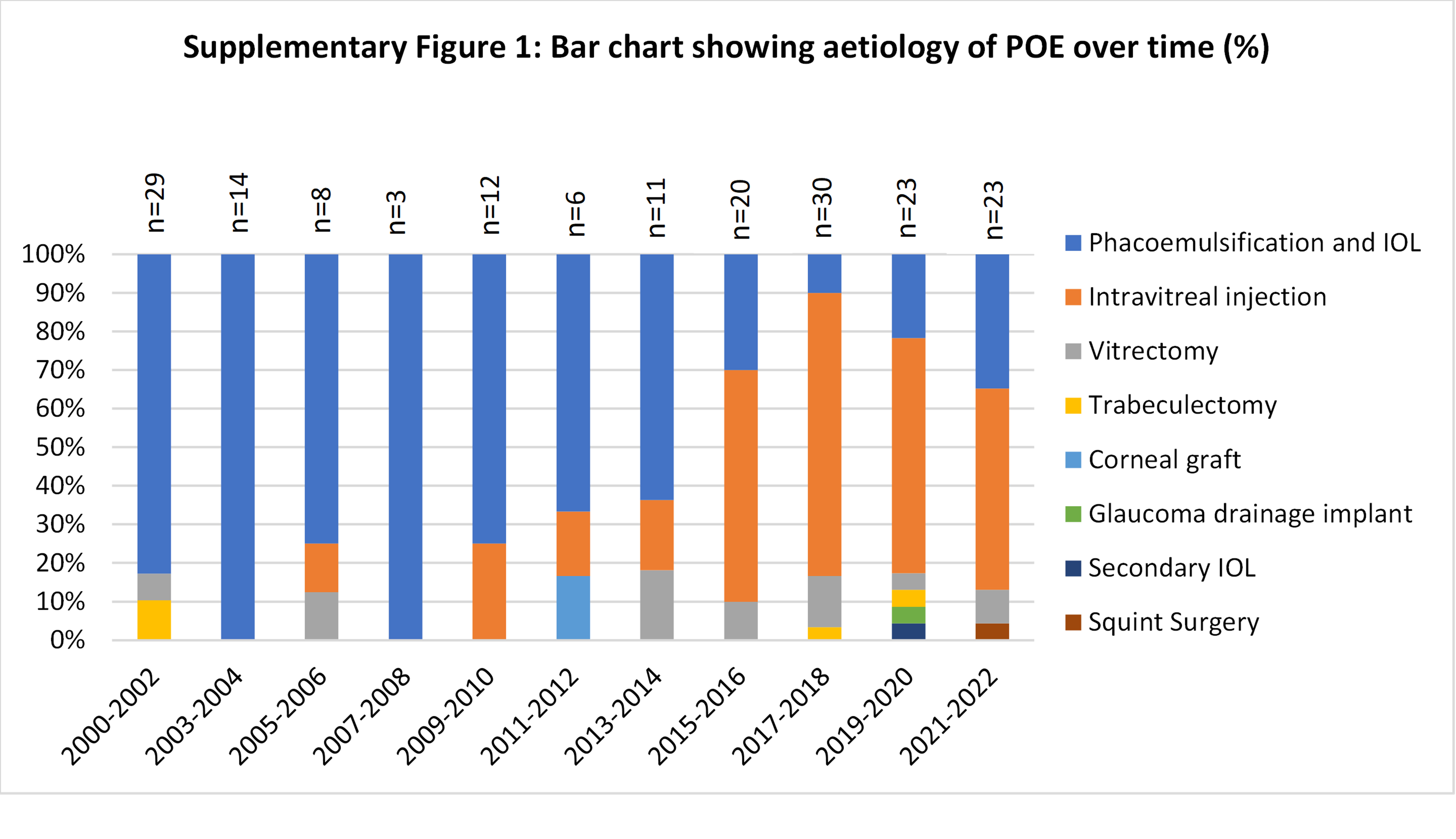

Supplement: Supplementary file 1 — Supplementary Figure 1 [file 41433_2025_3673_MOESM1_ESM.tif]
