# Supplementary material for: Long term analysis of microbiological isolates and antibiotic susceptibilities in acute-onset postoperative endophthalmitis: a UK multicentre study
Source: Eye (Lond). 2025 Feb 12;39(8):1470–5. doi: 10.1038/s41433-025-03673-w (PMC12089534; doi:10.1038/s41433-025-03673-w)
Supplement: Supplementary file 2 — Supplementary Table 1 [file 41433_2025_3673_MOESM2_ESM.docx]

**Supplementary Table 1: Microbiological sampling technique**

| **Microbiological sampling technique** | **Number (%)** |
| --- | --- |
| AC tap alone (without vitreous sample) | 10 (5.6) |
| AC tap + vitreous tap/biopsy | 85 (47.5) |
| AC tap + vitrectomy with vitreous sample | 7 (3.9) |
| Vitreous tap/biopsy alone | 66 (36.9) |
| Vitrectomy with vitreous sample | 11 (6.1) |
| Total | 179 (100) |

AC = anterior chamber
