# Supplementary material for: Long term analysis of microbiological isolates and antibiotic susceptibilities in acute-onset postoperative endophthalmitis: a UK multicentre study
Source: Eye (Lond). 2025 Feb 12;39(8):1470–5. doi: 10.1038/s41433-025-03673-w (PMC12089534; doi:10.1038/s41433-025-03673-w)
Supplement: Supplementary file 3 — Supplementary Table 2 [file 41433_2025_3673_MOESM3_ESM.docx]

**Supplementary Table 2: Subgroup analysis across three centres**

|  | **Gram-positive bacteria** | **Gram-negative bacteria** | **Total culture-positive eyes** |
| --- | --- | --- | --- |
| **Sunderland Eye Infirmary** | 59 (80.8%) | 14 (19.2%) | 73 |
| **Oxford Eye Hospital** | 13 (86.7%) | 2 (13.3%) | 15 |
| **Southampton General Hospital** | 13 (81.3%) | 3 (18.3%) | 16 |
| *X*^2^ = 0.29, p=0.87 | | | |
