# Supplementary material for: Long term analysis of microbiological isolates and antibiotic susceptibilities in acute-onset postoperative endophthalmitis: a UK multicentre study
Source: Eye (Lond). 2025 Feb 12;39(8):1470–5. doi: 10.1038/s41433-025-03673-w (PMC12089534; doi:10.1038/s41433-025-03673-w)
Supplement: Supplementary file 4 — Supplementary Table 3 [file 41433_2025_3673_MOESM4_ESM.docx]

**Supplementary Table 3: Poisson regression analysis of bacterial isolates over study period**

| **Microorganism** | **Coefficient** | **95% CI** | **p-value** |
| --- | --- | --- | --- |
| *Staphylococcus epidermidis* | 0.042 | -0.092 – 0.176 | 0.541 |
| Unspecified coagulase-negative *Staphylococci* | -0.029 | -0.157 – 0.098 | 0.651 |
| *Enterococcus faecalis* | -0.001 | -0.144 – 0.142 | 0.988 |
| *Pseudomonas aeruginosa* | -0.062 | -0.213 – 0.089 | 0.419 |
| *Staphylococcus aureus* | 0.009 | -0.143 – 0.162 | 0.903 |
| *Haemophilus influenzae* | -0.153 | -0.360 – 0.054 | 0.147 |
| *Serratia marcescens* | 0.007 | -0.202 – 0.216 | 0.949 |
| *Streptococcus spp.* | -0.010 | -0.144 – 0.124 | 0.881 |
| Other bacteria | -0.187 | -0.407 – 0.034 | 0.097 |

CI = confidence interval
