# Supplementary material for: Long term analysis of microbiological isolates and antibiotic susceptibilities in acute-onset postoperative endophthalmitis: a UK multicentre study
Source: Eye (Lond). 2025 Feb 12;39(8):1470–5. doi: 10.1038/s41433-025-03673-w (PMC12089534; doi:10.1038/s41433-025-03673-w)
Supplement: Supplementary file 5 — Supplementary Table 4 [file 41433_2025_3673_MOESM5_ESM.docx]

**Supplementary Table 4: Microbial yield of samples obtained.**

| **Intraocular sample** | **Number (%)** |
| --- | --- |
| Aqueous (n = 102) | 33 (32.4) |
| Vitreous (n = 169) | 94 (55.6) |
| Aqueous and/or vitreous (n = 179) | 104 (58.1) |
