# Supplementary material for: Long term analysis of microbiological isolates and antibiotic susceptibilities in acute-onset postoperative endophthalmitis: a UK multicentre study
Source: Eye (Lond). 2025 Feb 12;39(8):1470–5. doi: 10.1038/s41433-025-03673-w (PMC12089534; doi:10.1038/s41433-025-03673-w)
Supplement: Supplementary file 6 — Supplementary Table 5 [file 41433_2025_3673_MOESM6_ESM.docx]

**Supplementary Table 5: Univariable logistic regression for association with poor visual outcome**

| **Variables** | **Coefficient** | **Odds ratio** | **95% CI** | **p-value** |
| --- | --- | --- | --- | --- |
| Age | 0.010 | 1.010 | 0.984 – 1.037 | 0.456 |
| Gender | 0.030 | 1.031 | 0.550 – 1.934 | 0.924 |
| Primary ophthalmic intervention | 0.293 | 1.340 | 0.958 – 1.874 | 0.087 |
| Days since primary procedure | 0.003 | 1.003 | 0.964 – 1.044 | 0.870 |
| Culture positive | 1.063 | 2.895 | 1.476 – 5.679 | 0.002 |
| Gram-positive | 1.009 | 2.743 | 0.879 – 8.556 | 0.082 |
| Polymicrobial | 1.555 | 4.737 | 0.948 – 23.66 | 0.058 |
| Sensitive to empirical antibiotics | 0.482 | 1.620 | 0.613 – 4.276 | 0.330 |
| Presenting visual acuity (logMAR) | 1.646 | 5.189 | 2.425 – 11.10 | <0.001 |

CI = confidence interval; logMAR = logarithm of the minimum angle of resolution
