# Supplementary material for: Long term analysis of microbiological isolates and antibiotic susceptibilities in acute-onset postoperative endophthalmitis: a UK multicentre study
Source: Eye (Lond). 2025 Feb 12;39(8):1470–5. doi: 10.1038/s41433-025-03673-w (PMC12089534; doi:10.1038/s41433-025-03673-w)
Supplement: Supplementary file 7 — Supplementary Table 6 [file 41433_2025_3673_MOESM7_ESM.docx]

**Supplementary Table 6: Multivariable logistic regression for association with poor visual outcome**

| **Variables** | **Coefficient** | **Odds ratio** | **95% CI** | **p-value** |
| --- | --- | --- | --- | --- |
| Constant | -3.414 | 0.033 |  | <0.001 |
| Presenting visual acuity (logMAR) | 1.545 | 4.687 | 2.127 – 10.327 | <0.001 |

CI = confidence interval; logMAR = logarithm of the minimum angle of resolution
